# Cost-effectiveness of an oral cholate challenge test for the management of patients at risk for large esophageal varices

**DOI:** 10.1371/journal.pone.0313006

**Published:** 2024-11-22

**Authors:** Shailesh Chavan, Michael P. McRae, Kelly R. Pitts, Gregory T. Everson

**Affiliations:** 1 HepQuant, LLC, Denver, Colorado, United States of America; 2 Custom DX Solutions LLC, Houston, Texas, United States of America; Medical University of Graz: Medizinische Universitat Graz, AUSTRIA

## Abstract

**Aims:**

The dual oral cholate challenge test (DuO) quantifies liver function and portal-systemic shunting. Herein we report the economic impact of the use of the DuO Disease Severity Index (DSI) in the clinical management of patients with chronic liver disease suspected of having large esophageal varices.

**Methods:**

A Markov health state transition model of 100,000 patients with chronic liver disease suspected of having varices was populated with previously reported epidemiological, utility, and price data to assess the cost-effectiveness of employing the DuO test against the standard of care. The model examined the clinical and economic impact of healthcare management decisions all centered around the DSI score and given fixed prices of the DuO test.

**Results:**

In the target population, the combined strategy of healthcare management decisions based on DSI results would be highly cost-effective within two years for a price of $3,250 per DuO test. These same management decisions would save 2,740 lives over five years. For a price of ≤$3,213 per test, this intervention would be cost-saving within two years, and for ≤$4,100 per test it would be cost-saving within five years.

**Conclusions:**

Clinical decisions based on DSI from DuO are cost-effective in the management of patients with chronic liver disease suspected of having large esophageal varices. Future studies of direct comparison of DuO with other noninvasive tests are warranted. The DuO test offers a simplified approach that could enhance the clinical and research utility of liver function testing.

## Introduction

Chronic liver disease is one of the leading causes of death in the United States (US) accounting for over 51,000 deaths in 2020 [1]. Cirrhosis is the final common pathway for most chronic liver diseases and is a resource-intensive condition. In the US, over 500,000 cirrhosis hospitalizations occur each year [2], and roughly two-thirds of the patients who survive their hospitalization experience readmission at a cost of over $20,000 per occurrence [3]. In 2015, the medical costs for patients with cirrhosis in employer-sponsored insurance plans were over $9.5 billion [4], or approximately $20 billion for the entire US population [5].

The etiology of cirrhosis in the Americas is shifting from active hepatitis B virus and hepatitis C virus (HCV) infection towards resolved or treated viral hepatitis, alcohol consumption, and metabolic dysfunction-associated steatotic liver disease (MASLD). These data are in line with increases in obesity and alcohol consumption in the Americas [6]. The burden of cirrhosis remains substantial, owing to under-diagnosis and under-treatment of chronic liver disease, and the number of deaths and cases of decompensated cirrhosis (DC) are projected to rise in the next decade. Given the high prevalence and cost of chronic liver disease, there is an urgent need to identify early-stage disease and manage patients most at risk for progression.

Baveno VII guidelines recommend noninvasive methods for ruling out CSPH, using liver stiffness measurements (LSM) by transient elastography (TE) ≤15 kPa and platelet count (PLT) ≥150 × 10^3^ μL^-1^, and, ruling out large esophageal varices, using LSM <20 kPa and PLT >150 × 10^3^ μL^-1^ [7]. However, the accuracy of TE is operator-dependent and potentially compromised by overweight, obesity, hepatic inflammation, hepatic congestion, and metabolic dysfunction-associated steatohepatitis (MASLD/MASH). Additionally, PLT may be affected by non-hepatic factors. The consensus guidelines concluded that it is desirable to refine and validate surrogates for portal hypertension to identify risk for LEV particularly in patients with MASH, and to evaluate emerging methods, such as tests addressing liver function [7].

The dual oral cholate challenge test (DuO) test addresses this unmet need. Similar to creatinine clearance for the kidney, DuO is a minimally invasive test of liver function and physiology, utilizing the principles of hepatic clearance of cholate to provide a quantitative measure of hepatocellular uptake, hepatic perfusion, and portal-systemic shunting [8]. Research studies have demonstrated that DuO test parameters accurately risk stratify patients [9], predict progression [10], and monitor treatment response [11–13].

The DuO test generates a disease severity index (DSI) based on the clearance of an orally administered distinguishable cholate, a naturally occurring molecule endogenous to the human body that is labeled with nonradioactive isotopes, 13C or deuterium. DSI ≤18.3 has been validated in two US cohorts, HALT-C and SHUNT-V, as a cutoff for avoidance of endoscopy [9]. The same cutoff can be used to partition patients referred to endoscopy into good or stable function (DSI ≤18.3) and low risk for clinical outcome, or poor function (DSI >18.3) and higher risk for clinical outcome. Based on lower risk for clinical outcome, patients with DSI ≤18.3 may require less attention and, therefore, decreased resource utilization. Patients with DSI >18.3, whose risk for clinical outcome increases with increasing DSI, would require strict adherence to cirrhosis management guidelines.

In this study we evaluated the cost-effectiveness of the DuO test for use in adult patients with chronic liver disease who are considered at risk for varices, using a Markov health state transition model. We modeled the potential impact of the DuO results based on the intended uses of aiding the decision to avoid or proceed with screening endoscopy, prioritizing high-risk patients for more intense surveillance and treatment to prevent or delay decompensation, and reducing intensity of surveillance and treatment in low-risk patients to reduce costs and resource utilization. The model results are discussed and compared in the context of the standard of care (SOC).

## Methods

### Data

Data used in the economic model were derived from two previously published clinical studies: the Quantitative Liver Function Test (QLFT) ancillary study [14–17] of the Hepatitis C Antiviral Long-term Treatment Against Cirrhosis Trial (HALT-C) [18] and pivotal study, SHUNT-V [9]. Both studies were US multi-center studies conducted in hepatology clinics and are described below. The retrospective data were unidentifiable, did not involve interaction with living individuals, and cannot be linked to identifiable private information of living individuals.

#### HALT-C

HALT-C was a prospective, blinded, US multi-center study with prespecified esophagogastroduodenoscopy (EGD) and clinical endpoints. The QLFT protocol was an ancillary study of the HALT-C trial whose study design, subject eligibility, and primary results have been previously characterized [15–18]. The QLFT study enrolled 285 adult subjects with active chronic hepatitis C and advanced fibrosis or compensated cirrhosis. A total of 171 had Ishak fibrosis stages 2–4 and 114 had stages 5 or 6 (cirrhosis); 277 of 285 with baseline tests had analytical retesting of their samples by liquid chromatography/mass spectrometry (LC/MS), 220 were followed for up to 8 years for clinical outcomes, and 217 had EGD. Clinical outcomes included 2-point persistent increase in Child–Pugh score or development of ascites, encephalopathy, variceal hemorrhage (VH), or liver-related death. Hepatocellular carcinoma (HCC) as an outcome was considered separately. DSI 18.3 had >95% sensitivity, >99% negative predictive value, and negative likelihood ratio 0.08 for large esophageal varices (LEVs).

#### SHUNT-V

SHUNT-V was a prospective, blinded, multi-center US study designed to validate the diagnostic accuracy of DSI ≤18.3 from for ruling out LEVs [9]. Subjects were recruited between January 2019 and May 2021 via posted notices within the participating clinical centers. The study enrolled 306 subjects with 275 having both DSI and EGD, 238 of whom had Child–Pugh class A (CP-A) cirrhosis. Data from the patients with CP-A cirrhosis were combined with the HALT-C cohort (total n = 455) for the varices intended use.

### Cholate challenge test versions and test parameters

This economic model is based on DSI, a parameter equivalently defined from all cholate challenge test versions (HepQuant LLC, Denver, CO, USA) and is therefore applicable to DuO. The original SHUNT V1.0 involved the simultaneous administration of 13C-cholate by IV injection and d4-cholate orally and six timed peripheral venous blood samples at baseline and 5, 20, 45, 60, and 90 minutes. SHUNT V1.1 drops the 5-minute timepoint and SHUNT V2.0 uses only 20- and 60-minute timepoints. DuO is an oral-only version involving only the oral dose and two blood samples collected at 20 and 60 minutes. The DSI, included in all test versions, is an index from 0 (healthy) to 50 (severe disease) based on a patient’s cholate clearances relative to maximum cholate clearances in healthy controls. The DuO and SHUNT test versions have demonstrated equivalence in the determination of DSI [8].

### Patient characteristics

The HALT-C and SHUNT-V patient characteristics have been published previously [9] and are summarized here: mean age 56 ± 11 years, 61% male, mean BMI 32 ± 7 kg/m^2^, 84% White, 9% Black, and 13% Hispanic. Eighty-six percent of the subjects were overweight and 54% were obese. Of the 455 subjects, 177 had varices (38.9%), 128 were small (28.1%), and 49 were large (10.8%).

Across both the HALT-C (HCV) and SHUNT-V (mixed etiologies) studies, the etiologies of chronic liver disease included chronic hepatitis C (60.9%), MASLD/MASH (27.3%), alcoholic liver disease (8.1%), cryptogenic cirrhosis (3.5%), autoimmune hepatitis (3.7%), primary biliary cholangitis (0.4%), chronic hepatitis B (1.3%), and hemochromatosis (0.4%). A separate study evaluated DSI for prediction of esophageal varices and clinical outcome in 47 patients with primary sclerosing cholangitis [10].

Mean albumin was 4.0 ± 0.5 g/dL, alkaline phosphatase 100.9 ± 47.1 U/L, ALT 72.3 ± 73.4 U/L, AST 64.3 ± 52.2 U/L, bilirubin 0.8 ± 0.4 mg/dL, international normalized ratio (INR) 1.1 ± 0.1, and PLT 157,000 ± 68,000 μL^-1^. These results and the clinical scores (Model for End-Stage Liver Disease [MELD] 7.4 ± 1.9, CP 5.2 ± 0.4) suggested well-compensated liver disease.

The randomized HALT-C cohort had EGD and was followed for clinical outcome for up to 8 years. The SHUNT-V subjects had a single DuO test for the purpose of validating DSI ≤18.3 for ruling out LEVs and this cohort has not yet been studied for long-term clinical outcomes.

### Economic model description

A Markov health state transition model was developed to assess the cost-effectiveness of the DuO test (intervention arm) against the status quo (standard-of-care arm) for the management of patients with advanced chronic liver disease at risk for large varices. The base-case analysis assumed three management decisions, all centered on a DSI cutoff of 18.3, were taken at once, given a DuO test price of $3,250 (U.S. dollars):

Decision 1: Avoidance of endoscopy in patients with DSI ≤18.3
Decision 2: Reduction in clinical follow-up and testing in patients with DSI ≤18.3
Decision 3: Improved adherence to SOC guidelines for patients with DSI >18.3

An initial population of 100,000 patients with chronic liver disease suspected of having varices, and therefore being a potential candidate for endoscopy, was partitioned into subpopulations with no varices, small varices, large varices, and those with DC—and further partitioned by treatment status for large varices and the presence of variceal hemorrhage for small and large varices. The exit rates from the model consisted of liver-related mortality, procedure-related (EGD-related) mortality, and the all-cause mortality.

Each year, direct medical costs (comprising the cost of follow-up care and the cost of diagnostic tests) and health effects denominated in quality-adjusted life years (QALYs) accrued by the modeled population were calculated, and incident cases of DC and mortality (except the all-cause mortality) were output. Direct medical costs and health effects were discounted annually. Incremental cost-effectiveness ratio (ICER) was calculated as the ratio of the excess medical costs of the intervention arm to the QALYs gained by the intervention (relative to the SOC arm). The ICER was compared to a cost-effectiveness threshold shown in Table 1 and considered highly cost-effective if less than one times the cost-effectiveness threshold.

**Table 1:** Model population inputs and assumptions derived from internal analyses of the data from the QLFT ancillary study of HALT-C trial [14–17].

**Model inputs**			
**DuO test result in the initial population**			
% DSI >18.3	51% (30–70%)		
**DuO test sensitivity at DSI >18.3**			
Small varices	70.9%		
Large varices	93.4%		
**Population distribution**	**Overall**	**DSI >18.3**	**DSI ≤18.3**
No varices	66%	48%	84%
Small varices	24%	33%	14%
Large varices	10%	19%	1%
**Model assumptions**			
**Response to diagnosis in subjects with DSI ≤18.3**			
% Large varices treated	0%		
% DSI ≤18.3 receives intervention	0%		
**Response to diagnosis in subjects with DSI >18.3**			
% Large varices treated	100%		
% DSI >18.3 receives intervention	100%		
**Other parameters**			
Discount rate	3%		
Cost-effectiveness threshold for highly cost-effective intervention	$20,000 per QALY		

Abbreviations: DSI, disease severity index; QALY, quality-adjusted life year

The population structure, inputs to the health state transition model, and model parameters are shown in Tables 1–4. Stable patients undergoing endoscopy to check for varices will typically have underlying compensated CP-A cirrhosis or advanced fibrosis with low PLT or radiologic findings suggestive of cirrhosis or portal hypertension.

**Table 2:** Markov health state transition model inputs.

**Progression rate, annual**	**Overall**	**DSI >18.3**	**DSI ≤18.3**
No varices → DC	4.0%^a^	6.6%	2.0%^a^
Small varices → DC	8.3%^a^	11.9%	3.4%^a^
DSI ≤18.3 → DSI >18.3	-	-	2.5% (2.0–3.0%)
Large varices, not treated → DC	-	-	13.5%^a^
Large varices, treated → DC	13.7%^a^	13.5%^a^	
VH → Death	12.9%^a^		
VH, post-treatment → Death	-	12.9%^a^	
Re-bleeding → Death	12.9%^a^	-	-
DC → Death	12.9%^a^		
EGD → Death	0.001%^a^		
**Other**			
Reduction in progression to DC following intervention for DSI >18.3 population	35% (20–50%)		
**Population split, initial**	**Overall**	**DSI >18.3**	**DSI ≤18.3**
Small varices with VH	5%^a^		0.1%^a^
Large varices with VH	15%^a^		
Large varices, treated, with re-bleeding	2%^a^	-	-
Large varices, treated, with post-treatment VH	-	2%^a^	-

Abbreviations: DC, decompensated cirrhosis; DSI, disease severity index; EGD, esophagogastroduodenoscopy; UI, uncertainty interval; VH, variceal hemorrhage

^a^ UI of ±20% was assumed

**Table 3:** Annual healthcare and diagnostic costs, U.S. dollars [19].

**Healthcare costs**				
	**Without VH**	**With VH**	**Without rebleeding**	**With rebleeding**
**No varices**	145^a^	-	-	-
**Small varices**	155^a^	5,010^a^	-	-
**Large varices, treated**	1,110 (585–1,332)^b^		1,110 (585–1,332)^b^	5,010^a^
**Large varices, untreated**			-	-
**DC, subsequent years**	24,755 (4,690–29,706)^b^	-	-	-
**First year**	25,595 (16,430–30,714)^b^	-	-	-
**Diagnostic costs**				
EGD	2,642 (1,882–3,641)			
Standard lab tests, including alpha fetoprotein	475 ^a^, [20]			
Ultrasound surveillance for HCC	745 ^a^, [21]			

Abbreviations: DC, decompensated cirrhosis; EGD, esophagogastroduodenoscopy; HCC, hepatocellular carcinoma; UI, uncertainty interval; VH, variceal hemorrhage

^a^ UI of ±20% was assumed

^b^ Upper UI of +20% was assumed

**Table 4:** Quality-adjusted life years (QALY) inputs [22] in life-years. An uncertainty interval of ±20% was assumed.

	Without VH	With VH	Without rebleeding	With rebleeding
No varices	0.92 (0.72–1.00)	-	-	-
Small varices	0.79 (0.77–0.81)	0.69 (0.44–0.69)	-	-
Large varices, treated	0.83 (0.79–0.87)	0.69 (0.44–0.69)	0.83 (0.79–0.87)	0.69 (0.44–0.69)
Large varices, not treated	0.76 (0.70–0.79)	0.69 (0.44–0.69)	-	-
Decompensated cirrhosis	0.69 (0.44–0.69)	-	-	-

Abbreviations: VH, variceal hemorrhage

For the base-case analysis under the SOC arm, all patients suspected of having varices received an initial EGD. If no varices were detected, an EGD was repeated every two years; if small varices were detected, once yearly; if large varices were detected, twice yearly. Standard laboratory tests, including alpha-fetoprotein and ultrasound surveillance for HCC, were also applied twice yearly.

**Fig 1:**
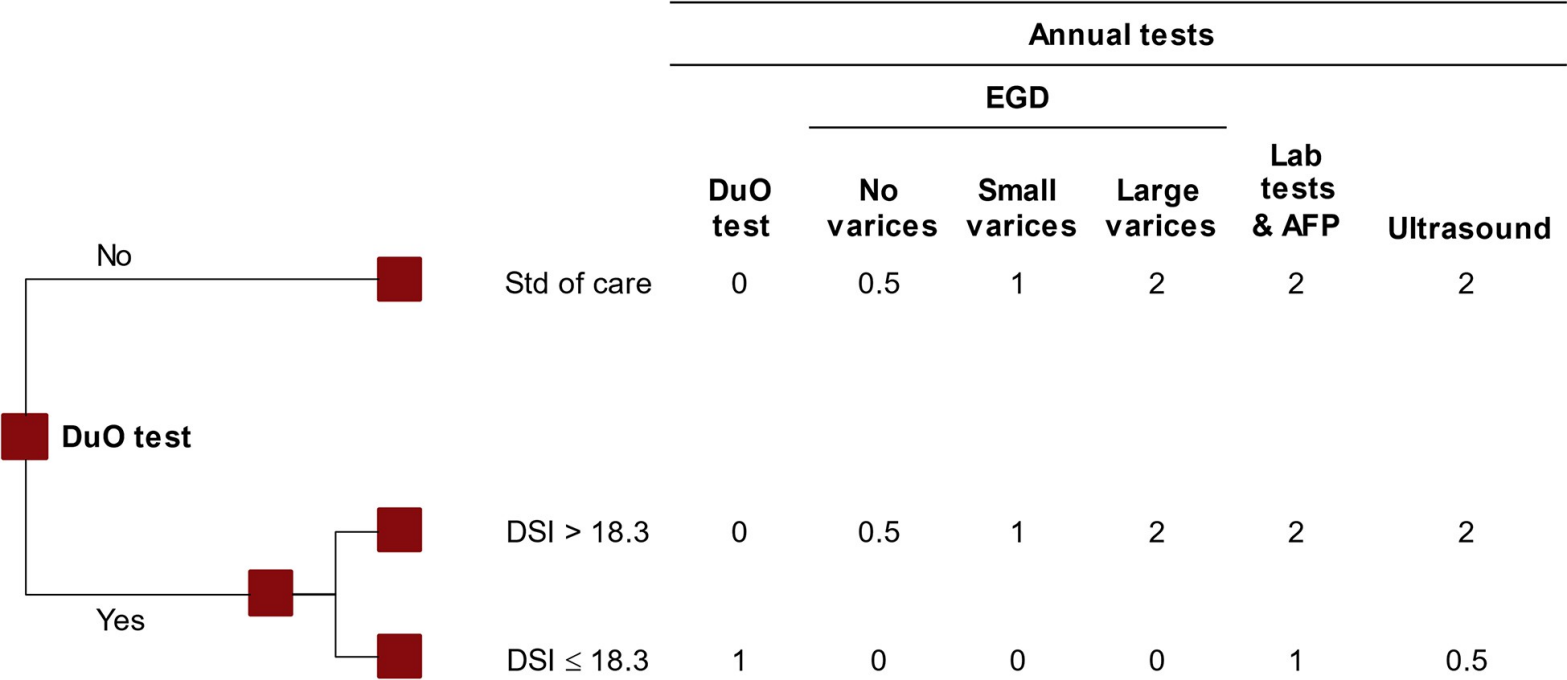
Testing schedules of standard of care and intervention arms. Abbreviations: EGD, esophagogastroduodenoscopy; Std of care, standard of care; DSI, disease severity index; AFP, alpha-fetoprotein.

For the base-case analysis under the intervention arm, all patients suspected of having varices received an initial DuO test. For patients with DSI >18.3, the testing schedule of the SOC arm described above was repeated. For those with DSI ≤18.3, the DuO test and standard laboratory tests, including for alpha-fetoprotein, were repeated once yearly, and ultrasound surveillance for HCC was repeated every two years. The testing decisions corresponding to the modeled intervention arms are summarized in **Fig 1**.

### Uncertainty analysis

All uncertain variables were assumed to be betaPERT-distributed, with uncertainty intervals (UIs) shown in Tables 1–4. The betaPERT is a special case of the beta distribution that is defined by three parameters: the minimum, the likeliest value, and the maximum.

First, a one-way sensitivity analysis was conducted to identify the variables contributing to the highest uncertainty in the number of years necessary for the intervention arm to become highly cost-effective relative to the SOC.

Then, a what-if analysis varying the price of a DuO test with respect to the number of years necessary for attaining a highly cost-effective intervention was conducted.

Lastly, (1) since DSI quantifies liver function and has been linked to the risk for clinical outcome and (2) since DSI >18.3 defines a “higher risk” patient (one at risk for large esophageal varices and its consequences, as well as risk for clinical outcome) and DSI >18.3 determination would increase adherence to clinical follow-up and reduce the rate of decompensation, the analysis was further segmented by management decision (avoidance of endoscopy in patients with DSI ≤18.3, reduction in clinical follow-up and testing in patients with DSI ≤18.3, improved adherence to SOC guidelines for patients with DSI >18.3) to isolate the economic impact of individual decisions.

In all cases, break-even prices (the price at which the intervention was cost-neutral relative to the SOC) at two and five years were reported.

## Results

### Base-case analysis

The base case, including the impact of all three clinical management decisions related to DSI 18.3 for a modeled cohort of 100,000 patients, resulted in 3,498 incident cases of DC averted in the intervention arm relative to the SOC as well as 2,740 lives saved—with 4,342 QALYs gained over five years. Over the same period, a total of $236 million (M) in direct medical costs was saved. Finally, the intervention was cost-effective within two years and cost-saving within three years, with $74M in direct medical costs averted relative to the SOC over the three-year period.

### Uncertainty–one-way sensitivity analysis

One-way sensitivity analysis revealed that eight uncertain variables (excluding the price) accounted for greater than 80% variance in the number of years necessary for a highly cost-effective intervention: (1) reduction in progression to DC following intervention for DSI >18.3 population, (2) healthcare cost, DC, subsequent years, without VH, (3) % DSI >18.3, (4) progression rate, no varices → DC, overall, (5) healthcare cost, ultrasound surveillance for cancer, (6) diagnostic cost, EGD, (7) QALY utility, no varices, without VH, (8) healthcare cost, DC, first year, without VH.

### Uncertainty–what-if analysis

**Fig 2:**
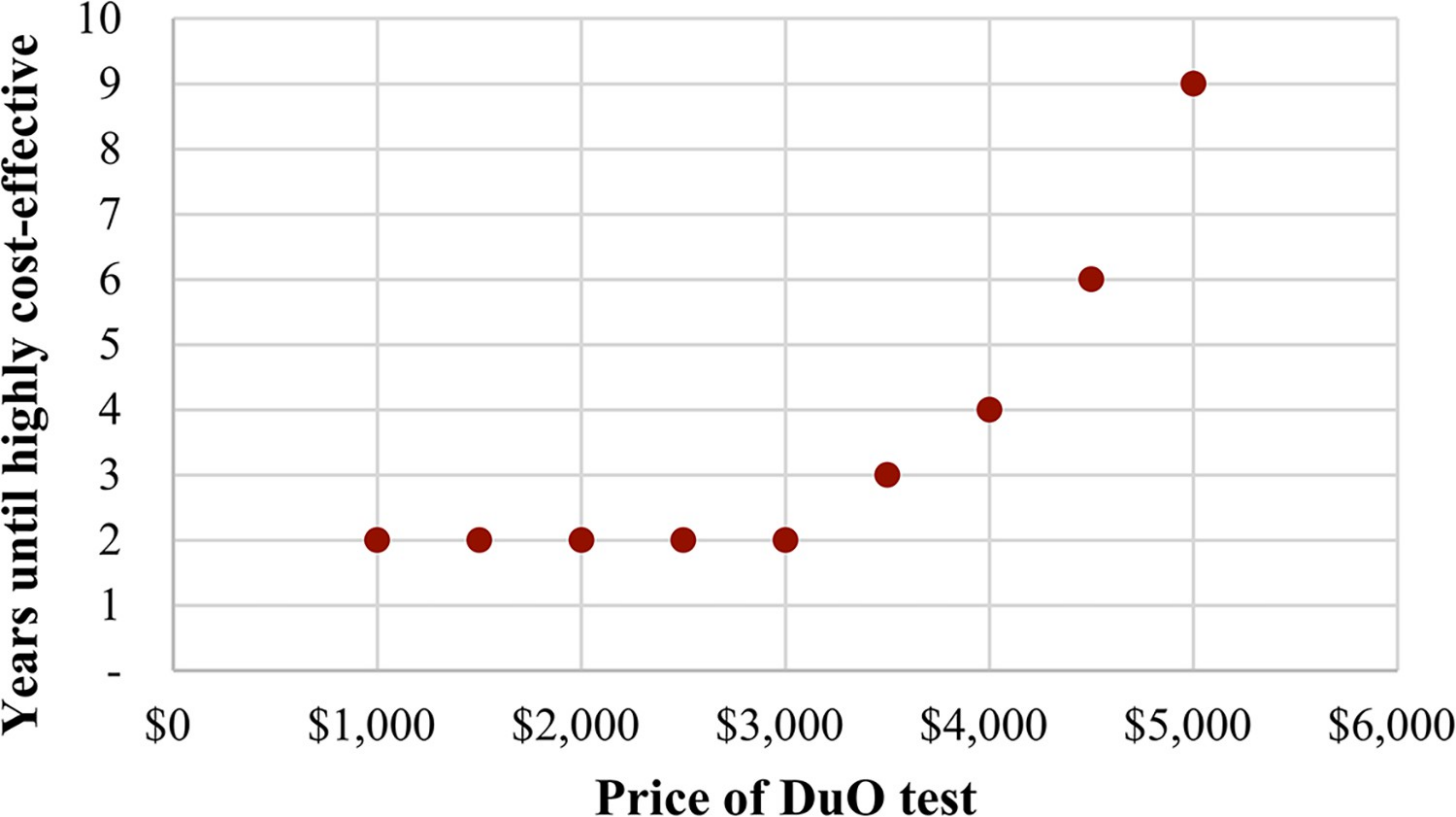
What-if analysis for price of DuO test for all three management decisions.

Maintaining all three management decisions described above, a what-if analysis with respect to the price of the DuO test revealed that the timing of achieving a highly cost-effective intervention, relative to SOC, varied from two years for a test price of $1,000 to nine years for a price of $5,000, as shown in **Fig 2**. At a price of ≤$3,213 per DuO test, this intervention would be cost-saving within two years; at ≤$4,100 per test, it would be cost-saving within five years.

Next, examining the individual impact of the three individual management decisions for the same cohort of 100,000 patients revealed that (1) avoidance of endoscopy in patients with DSI ≤18.3 alone would be cost-saving within two years at a DuO test price of ≤$1,447, and within five years at a price of ≤$1,409; (2) reduction in clinical follow-up and testing in patients with DSI ≤18.3 alone would be cost-saving within two years at a price of ≤$1,488, and within five years at a price of ≤$1,436; and (3) improved adherence to SOC guidelines for patients with DSI >18.3 would be cost-saving within two years at a price of ≤$1,541, and within five years at a price of ≤$1,161. The latter healthcare management decision alone (decision 3) would see 3,498 incident cases of DC and 2,737 deaths relative to SOC averted, with 4,339 QALYs gained over a five-year period.

## Discussion

The cholate challenge tests of liver function and physiology generate a score of liver health, the Disease Severity Index (DSI). DSI has demonstrated excellent diagnostic performance over a wide array of clinical studies and has been correlated with fibrosis stage [15], portal hypertension [23], varices [9, 14, 24–26], clinical outcome [17, 27, 28], and hepatic improvement after sustained virological response [16]. Additionally, its exceptional within-individual reproducibility [29, 30] makes DSI useful for detecting treatment effects, for example, in therapies for MASLD/MASH [31] and portal hypertension [32]. The economic model reported herein evaluated the cost-effectiveness of DSI results in the management of patients with chronic liver disease at risk for esophageal varices.

DSI is the main output for all versions of the cholate challenge test. The initial test version (SHUNT V1.0) required intravenous injection and six blood samples over 90 minutes, presenting practical limitations. The DuO version simplifies test collection with the oral-only administration of cholate and two blood samples at 20 and 60 minutes. Furthermore, the DSI from the DuO is highly reproducible [30] and equivalent [8] to DSI from SHUNT versions, making an economic model based on DSI applicable to all test versions. By simplifying test collection, DuO has the potential to broaden the utility of function testing in clinical practice.

DSI is highly correlated with increasing probability of LEVs (*p*<0.001) [9]. Knowing the likelihood that a given DSI level is associated with a particular risk of LEVs is highly relevant to clinical decision-making. At a low DSI and low probability of LEVs, the clinician may consider delaying endoscopy. At a high DSI and high risk of LEVs, an urgent endoscopy may be needed.

Markov health state transition modeling found that, within a period of two years, the combined strategy of healthcare management decisions based on DSI would be highly cost-effective for a price of $3,250 per DuO test and would save 2,740 lives by the fifth year. The model also assumed that patients with DSI >18.3 receive SOC follow-up; but, in addition, we factored better triaging and adherence to SOC guidelines for DSI >18.3, resulting in reduction in rate of decompensation and hospitalization. In our analysis, we included reduction in procedure-associated mortality for DSI ≤18.3, but the main impact of avoidance of endoscopy due to DSI ≤18.3 was reduction in overall cost. Although clinical risks and costs related to variceal hemorrhage for missed varices in patients with DSI ≤18.3 were included in the model, these effects were completely offset by the reduction in clinical follow-up. The latter effect also lowered costs. Each of these improvements to healthcare management decisions contributed equally to the consideration of a DuO test price.

The current model is a decision-scientific tool that allows measuring the impact of healthcare management decisions, so the uncertainty analysis included the healthcare management decision dimension, whereby the impact of all three decisions at once, as well as individually, was assessed.

While we acknowledge the Baveno VII recommendations for the use of LSM and PLT for ruling out CSPH and LEVs [7], the direct comparison of DuO to Baveno criteria was not an objective of this study. Interestingly, we found in our US-based pivotal study (SHUNT-V) that LSM was used for the endoscopy decision in only 86 out of 275 (31%) patients. A potential reason for this is that the accuracy of FibroScan may be compromised in overweight/obese patients, which now represents the patient population that is undergoing screening endoscopy for varices in the United States. This finding supports the use of alternative methods, such as HepQuant DuO, to more accurately define large varices risk in the population.

In the SHUNT-V study, we compared DSI (n = 275) to LSM (n = 86) in ruling out any varices across clinically relevant ranges for DSI, kPa, and PLT [9]. We found that the addition of a PLT cutoff to the DSI cutoff avoids more EGDs than DSI cutoff alone without compromising sensitivity or miss rate (DSI <25 with PLT >150,000 μL^-1^ would have avoided 33% of EGDs; DSI <25 with PLT >110,000 μL^-1^ would have avoided 46% of EGDs). Rates of EGD avoidance were similar at DSI 21–22 and kPa 18–20, but kPa 18–20 missed 10 to 15% more varices than DSI 21–22 at every PLT cutoff between 110,000 to 150,000 μL^-1^. Although not a complete paired analysis of DSI and LSM, these preliminary results support the use of HepQuant DuO in aiding the endoscopy decision.

According to the Baveno VII consensus, treatment with non-selective beta blocker (NSBBs) should be considered for prevention of decompensation in patients with CSPH, as such patients may not need a screening endoscopy for the detection of varices [7]. However, in our pivotal SHUNT-V study of US hepatology practices, only a third of the study subjects had kPa by FibroScan performed within a year of the endoscopy. This suggests that the Baveno VII recommendation, while implemented in Europe, is currently less frequently applied in the US. Perhaps one reason for hepatologists in the United States to use NSBB therapy more selectively is due to the concern for potential side effects, some of which may be associated with considerable morbidity and mortality. In addition, the only method for detecting a positive therapeutic effect of NSBB is by performance of hepatic venous pressure gradient (HVPG), which is expensive, invasive, and impractical in clinical practice.

Current EASL and AASLD guidelines suggest empirically treating patients with NSBBs if portal hypertension is suggested by noninvasive testing. This recommendation is not currently universally accepted, but its implementation is evolving. A recent AASLD publication highlights the advantages and disadvantages of noninvasive testing for portal hypertension and suggested that HepQuant could be useful for this assessment [33]. In the HepQuant studies there was no direct comparison with other noninvasive tests, limiting the ability to assess the impact of other noninvasive testing on our economic model.

One additional limitation of the current model is that it does not account for the fraction of small varices cases treated with NSBBs that would not undergo repeat EGD testing. However, in SHUNT-V we found that a small percentage of small varices (5 out of 76 [6.6%]) were treated (all had NSBB therapy) and, therefore, exclusion of such patients from repeat EGD would have minimal impact on the model results.

Liver disease is typically silent and undetected until the late stages of the disease. Given the high prevalence of fatty liver disease in the overweight and obese population, additional tools beyond elastography and standard laboratory tests are needed. One potential reason for underutilization of current testing might be uncertainty in the accuracy of elastography in the overweight and obese population with fatty liver disease. In addition, the initial costs and training requirements for transient elastography may hinder its adoption in primary care and GI offices. The DuO test has utility across many etiologies of liver disease and is not directly affected by body habitus. Given these factors, the DuO may find greater applicability beyond the initial intended uses in the US population. Due to simplicity of test collection, DuO has the potential for broad application, in both clinical settings and research settings.

## Conclusions

In summary, the DuO test is an easy to administer, minimally invasive test yielding a DSI that allows for improved healthcare decision-making, with significant cost saving and reduced morbidity and mortality in the immediate future for patients with chronic liver disease suspected of having varices. This independent health economic analysis revealed that the DuO test, at a price per test of $3,250, in patients with chronic liver disease suspected of having varices would be highly cost-effective within two years. Over five years, DuO testing could save $236M in direct medical costs for a cohort of 100,000 patients with CLD due to reduction in EGDs as well as clinical follow-up and testing in those with DSI ≤18.3 and improved adherence to SOC guidelines for DSI >18.3 patients.
